# Anti-N-Methyl-D-Aspartate-Receptor Encephalitis: A 10-Year Follow-Up

**DOI:** 10.3389/fpsyt.2020.00245

**Published:** 2020-05-15

**Authors:** Sophie Meixensberger, Ludger Tebartz van Elst, Tina Schweizer, Simon J. Maier, Harald Prüss, Bernd Feige, Dominik Denzel, Kimon Runge, Kathrin Nickel, Miriam Matysik, Nils Venhoff, Katharina Domschke, Horst Urbach, Evgeniy Perlov, Dominique Endres

**Affiliations:** ^1^Section for Experimental Neuropsychiatry, Department of Psychiatry and Psychotherapy, Medical Center – University of Freiburg, Faculty of Medicine, University of Freiburg, Freiburg, Germany; ^2^Department of Psychiatry and Psychotherapy, Medical Center – University of Freiburg, Faculty of Medicine, University of Freiburg, Freiburg, Germany; ^3^Department of Neurology and Experimental Neurology, Charité – Universitätsmedizin Berlin, Berlin, Germany; ^4^German Center for Neurodegenerative Diseases (DZNE), Berlin, Germany; ^5^Department of Rheumatology and Clinical Immunology, Medical Center – University of Freiburg, Faculty of Medicine, University of Freiburg, Freiburg, Germany; ^6^Centre for Basics in Neuromodulation, Faculty of Medicine, University of Freiburg, Freiburg, Germany; ^7^Department of Neuroradiology, Medical Center – University of Freiburg, Faculty of Medicine, University of Freiburg, Freiburg, Germany; ^8^Clinic for Psychiatry Luzern, St. Urban, Switzerland

**Keywords:** anti-N-methyl-D-aspartate-receptor encephalitis, catatonia, antibodies, follow-up, long-term, neuroleptics, psychotherapy

## Abstract

**Background:**

Anti-N-methyl-D-aspartate-receptor (NMDA-R) encephalitis is an autoimmune disease of the brain first described in 2007. The aim of this paper is to present a 10-year follow-up case history.

**Case Presentation:**

The authors present the case of a 39-year-old female patient who developed an anti-NMDA-R encephalitis in 2009 with predominant severe catatonic symptoms. Anti-inflammatory therapy led to the disappearance of catatonic symptoms and was discontinued during the course of the disease. After acute therapy, the patient achieved an almost full recovery presenting with ongoing discrete symptoms of sensory overload, subtle cognitive deficits, and fatigue/reduced energy levels. The follow-up investigation in 2019 showed inconspicuous findings in laboratory diagnostics and magnetic resonance imaging. Electroencephalography (EEG) analysis using independent component analysis detected left hemispherical spike-wave complexes and intermittent slowing. Regarding the sensory overload and reduced energy level, the patient benefited from low-dose neuroleptics (risperidone, amisulpride). In terms of sensory overload associated with experiences of panic, cognitive deficits and coping with the disease, she improved with cognitive behavioral therapy (CBT).

**Conclusion:**

Anti-inflammatory treatment led to almost full recovery with persistent disappearance of catatonic symptoms; however, a dysexecutive syndrome led to ongoing relevant problems with good response to low-dose atypical neuroleptics and CBT. The patient had persistent EEG alterations that indicated continuing neuronal network instability. Therefore, the case demonstrates the importance of multidisciplinary outpatient treatment following acute therapy for anti-NMDA-R encephalitis in patients with ongoing psychiatric deficits. For the symptomatic treatment of executive dysfunctions, “classical” psychiatric treatment may be helpful in the course of the disease.

## Background

Anti-N-methyl-D-aspartate-receptor (NMDA-R) encephalitis is a neuroinflammatory disease first identified in 2007 (1). It is mainly associated with cerebrospinal fluid (CSF) immunoglobulin G (IgG) autoantibodies against the GluN1 subunit of the NMDA-R (2, 3). Predominantly children and young adults (median age 21 years), more frequently females, are affected (4). Originally, the disease was described in association with ovarian teratomas (1, 5). Apart from malignancies, herpes simplex encephalitis is a confirmed trigger of anti-NMDA-R encephalitis (2, 6). The clinical pattern often begins with low-grade fever, malaise, headache, or mood changes (7, 8), followed by a subacute phase with changes in behavior, cognitive deficits, and psychiatric symptoms, including delusions, hallucinations, and catatonia, speech disorders, and often seizures (7, 8). Further neurological complications, such as movement abnormalities, dyskinesias or rigidity, dysautonomia, and a decreased level of consciousness, typically develop later in the course of the disorder (7, 8). About one month after disease onset, anti-NMDA-R encephalitis typically presents with an extreme overlap of diverse neuropsychiatric symptoms; only about 5% of patients display a monosymptomatic course (4). Affected patients usually respond well to anti-inflammatory treatment, but psychiatric symptoms, such as disinhibition, impulsivity, and sleep disturbances, may persist over months to years (2).

**Rationale:** Little is known about the long-term course and treatment of ongoing psychiatric deficits in anti-NMDA-R encephalitis as it is a relatively new clinical pattern. However, being the most frequently recognized autoimmune encephalitis in the last decade, it is of high clinical relevance (2). Therefore, the aim of the paper is to present one of the longest follow-up reports in the literature to date.

## Case Presentation

The authors present the follow-up of a 39-year-old female patient who developed anti-NMDA-R encephalitis in 2009 with a long course of disease (21 months) up to diagnosis (9). The initial findings of this patient have already been published as a case report (9). Before the onset of neuropsychiatric symptoms in 2009, the patient had always been mentally healthy and had worked as a business controller (9).

### Clinical and Treatment Course

**Figure 1:**
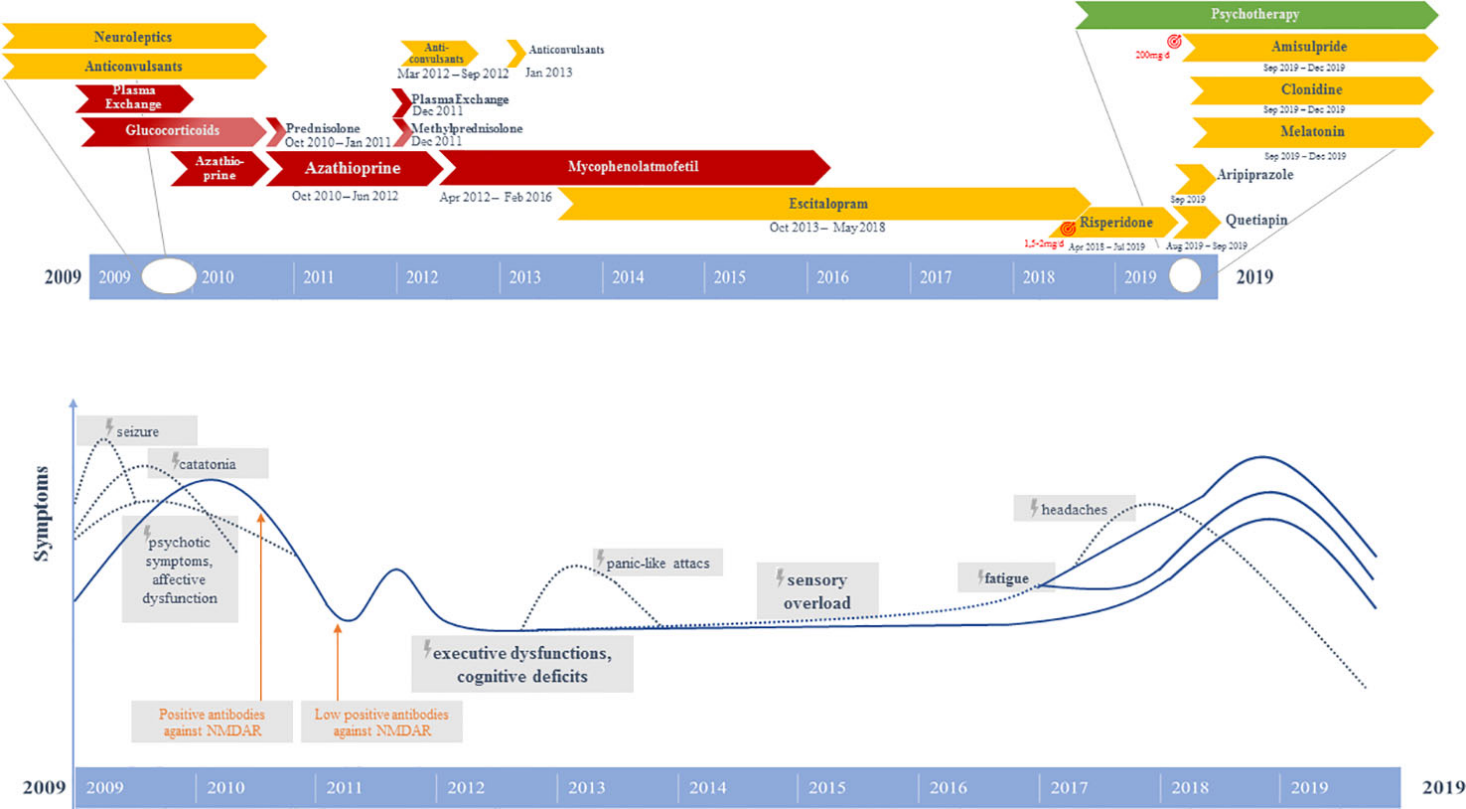
Graphical representation of the clinical course and therapeutic procedures. Above: Different pharmacological strategies. Below: Symptom course.

Initially, the patient presented a wide spectrum of symptoms including severe catatonia, delusions, cognitive deficits, as well as one epileptic seizure and states of altered consciousness (9). Evidence of anti-NMDA-R encephalitis came from the positive anti-NMDA-R IgG antibodies, hypoglutamatergic state in the left prefrontal cortex in the magnetic resonance spectroscopy (MRS), left hemispheric hypometabolism demonstrated in [^18^F]-fluorodeoxyglucose positron emission tomography (FDG-PET), and electroencephalography (EEG) alterations. The independent component analysis (ICA) of the EEG initially revealed three components with EEG slowing (9). The combination of 1) rapid onset of psychiatric symptoms/cognitive dysfunction, mutism, one seizure, catatonia, and states of altered consciousness; 2) EEG (slowing) and CSF (pleocytosis initially) pathologies; and 3) the detection of clearly positive IgG anti-GluN1 antibodies would also allow the syndrome diagnosis of anti-NMDA-R encephalitis, according to present criteria (3). Unfortunately, the currently recommended CSF testing or a confirmatory analysis in serum with another method was not performed at that time (3). Anti-inflammatory therapy (glucocorticoids, plasmapheresis) resulted in relevant clinical improvement with disappearance of the catatonic and delusional symptoms in parallel to a remarkable normalization of the FDG-PET (9). Since symptom onset in 2009, the patient had been unable to remember any dreams. Moreover, she had developed amnesia for initial symptoms. Following discharge from the psychiatric department in October 2010, the patient received a maintenance therapy consisting of prednisolone (40 mg/day, with gradual reduction of 5 mg/week; from 10 mg/day onwards gradual reduction of 2 mg/week) and maintenance therapy with azathioprine (100 mg/day). Prednisolone was fully tapered in January 2011. During aftercare in a rehabilitation clinic a relapse of psychiatric symptoms with thought disruptions, attention/concentration and memory disorders, irritability and insomnia occurred. Therefore, in December 2011 a second cycle of plasmapheresis followed by methylprednisolone pulse therapy (500 mg intravenously for 4 days) were performed and led to a slight improvement. Immunosuppression with azathioprine (100 mg/day) was continued until February 2012. However, it had to be discontinued due to the development of cholestatic hepatitis. Between April 2012 and February 2016, the patient received mycophenolate mofetil (MMF, 2000 mg/day). In 2016, the anti-inflammatory treatment was stopped. In the period between March 2012 and September 2012, the patient received a prophylactic network stabilizing treatment with levetiracetam (2000 mg/day). Beginning in September 2012, the patient had suffered from newly occurring panic-like attacks and the feeling of anxiety arising from a visual and acoustic sensory overload (especially when being in groups of people). Therefore, in January 2013, again a treatment attempt with levetiracetam (2000 mg/day for 3 weeks) for mood stabilization and prophylactic neuronal network stabilization was performed again reducing the frequency of attacks; but without sufficient symptom control. EEG and video-monitoring twice (in January and March 2013) as well as magnetic resonance imaging (MRI) revealed negative findings in terms of an epileptogenic focus. In June 2013, treatment with escitalopram (5 mg/day) was started with discrete positive effects in terms of improving the feeling of panic and anxiety. However, over the following years, sensory overload, cognitive dysfunctions, and fatigue continued. In February 2017, sensory overload phenomena worsened and holocephalic oppressive headaches were reported (visual analog scale: 4/10). In the course of 2018, sensory overload further increased, and the fatigue symptoms were accompanied by a reduction in energy levels. Therefore, a therapeutic attempt with low-dose risperidone (up to 2 mg/day) was started, which was clearly efficient in terms of reducing the sensory overload symptoms. The patient regained the ability to go to the cinemas, and the reduced energy level normalized, however, fatigue remained. About 1 year later, there was strong deterioration of energy levels and fatigue, which only improved after discontinuation of risperidone. However, as a result the sensory overload also reemerged strongly, and insomnia occurred. She suffered both from a reduced ability to fall asleep and to maintain sleep throughout the night. The sleep disorder was successfully treated with melatonin (4 mg/day). Quetiapine (50 mg/day) had no convincing sleep-inducing effect. In addition, a treatment with aripiprazole (2.5 mg/day) was started to increase the patient’s energy and reduce sensory overload. Even at a dose of 2.5 mg/day, an agonizing inner and psychomotor restlessness was evident, so aripiprazole was quickly discontinued. Finally, the sensory overload and reduced energy levels could be successfully improved with low-dose amisulpride (200 mg/day). High blood pressure was normalized with clonidine (225 µg/day), possibly contributing additionally to the reduction of sensory overload. In the cognitive behavioral therapy (CBT) sessions, states of sensory overload associated with experiences of panic were treated by a) identifying and reducing the stressful stimuli and b) challenging and changing dysfunctional cognitions and behaviors, improving emotion regulation and applying more adaptive coping strategies (e.g., mindfulness-based tasks against catastrophic thinking and to decrease high levels of stress). Furthermore, cognitive deficits were treated by cognitive training as well as acceptance and commitment therapy (ACT). In summary, initial anti-inflammatory therapy (glucocorticoids, plasmapheresis, azathioprine, MMF) as well as later low-dose neuroleptics (risperidone, amisulpride) and CBT had a remarkable impact on symptom relief (**Figure 1**).

### Main Syndrome Over the Last 10 Years

Since acute therapy and continuing to the present time, the patient has suffered mainly from an ongoing dysexecutive syndrome with 1) intense sensory overload, 2) cognitive deficits, and 3) reduced energy levels and fatigue:

1. The sensory overload sensitivity was triggered mainly by acoustic and visual stimuli and was accentuated in situations with many other people present. Thus, the patient was not able to visit markets or cinemas. Often, she experienced panic in such sensory overload situations, so that a panic disorder was assumed and an epileptic cause was ruled out.
2. The cognitive deficits consisted mainly of attention and concentration deficits and allowed the patient to only work a maximum of 1-2 hours per day. In addition, she has reported formal thought disorder with lapses of thoughts/thought blocking and a slowed but coherent train of thought.
3. Reduced energy levels and fatigue: She experienced ongoing fatigue symptoms (e.g., she always needed a nap at noon), therefore, only part-time employment was possible. Over the course of 10 years, episodes with reduced energy levels, in which the motivation to everyday actions was difficult for her, repeatedly evolved. She reported no overt depression, abulia, or anhedonia.

### Diagnostic Follow-Up Findings

**Figure 2:**
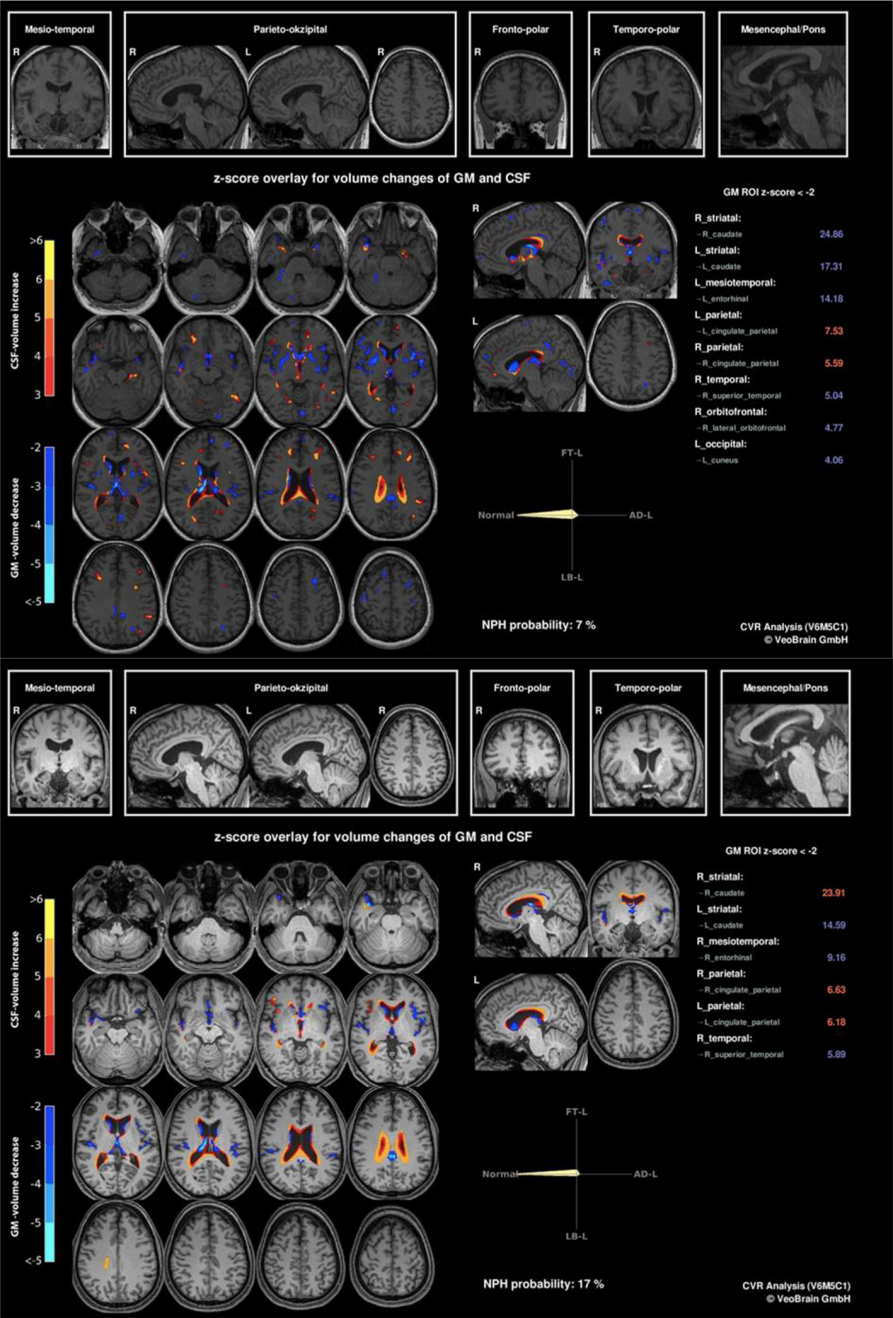
Initial (2010, at the top) and follow-up (2019, at the bottom) magnet resonance imaging findings with combined volume- and region-based analysis method (CVR) revealed an enlargement of the lateral ventricles with emphasis on the posterior horns and slight striatal and insular atrophy. There was no relevant change in findings between 2010 and 2019 (https://www.veobrain.com/?page=veomorph).

**Figure 3:**
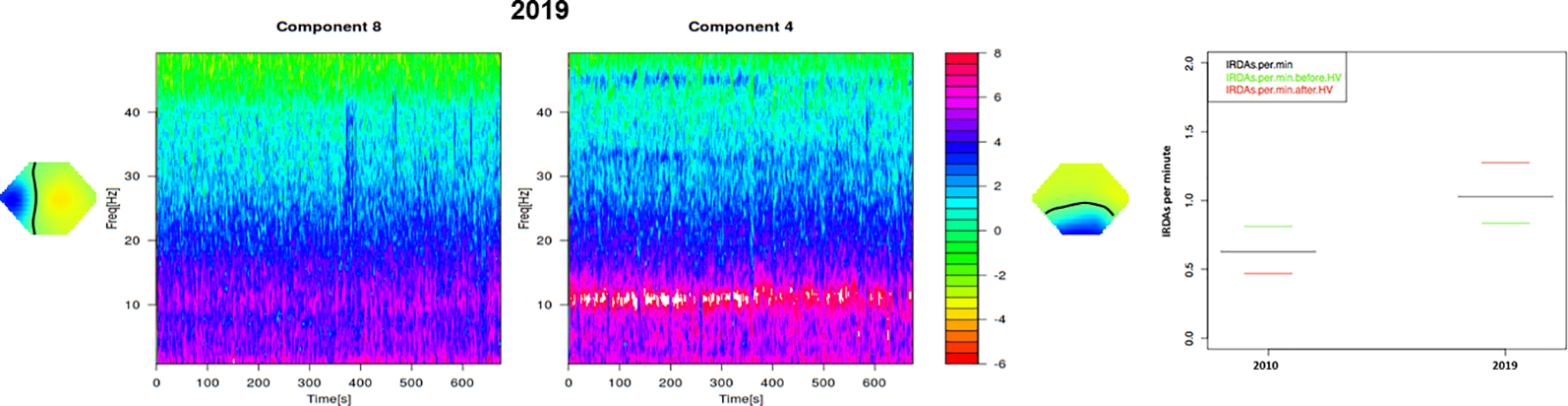
Electroencephalography (EEG) analysis using independent component analysis (ICA, left) and intermittent rhythmic delta activity (IRDA) rates (right). The follow-up EEG analysis in 2019 showed strong α-activity and some IRDAs in component 4 and 8 (left). The left-temporal component (8) contains individual spike wave complexes. Overall, there was an increase in IRDA rates from 2010 to 2019 (right).

**Figure 4:**
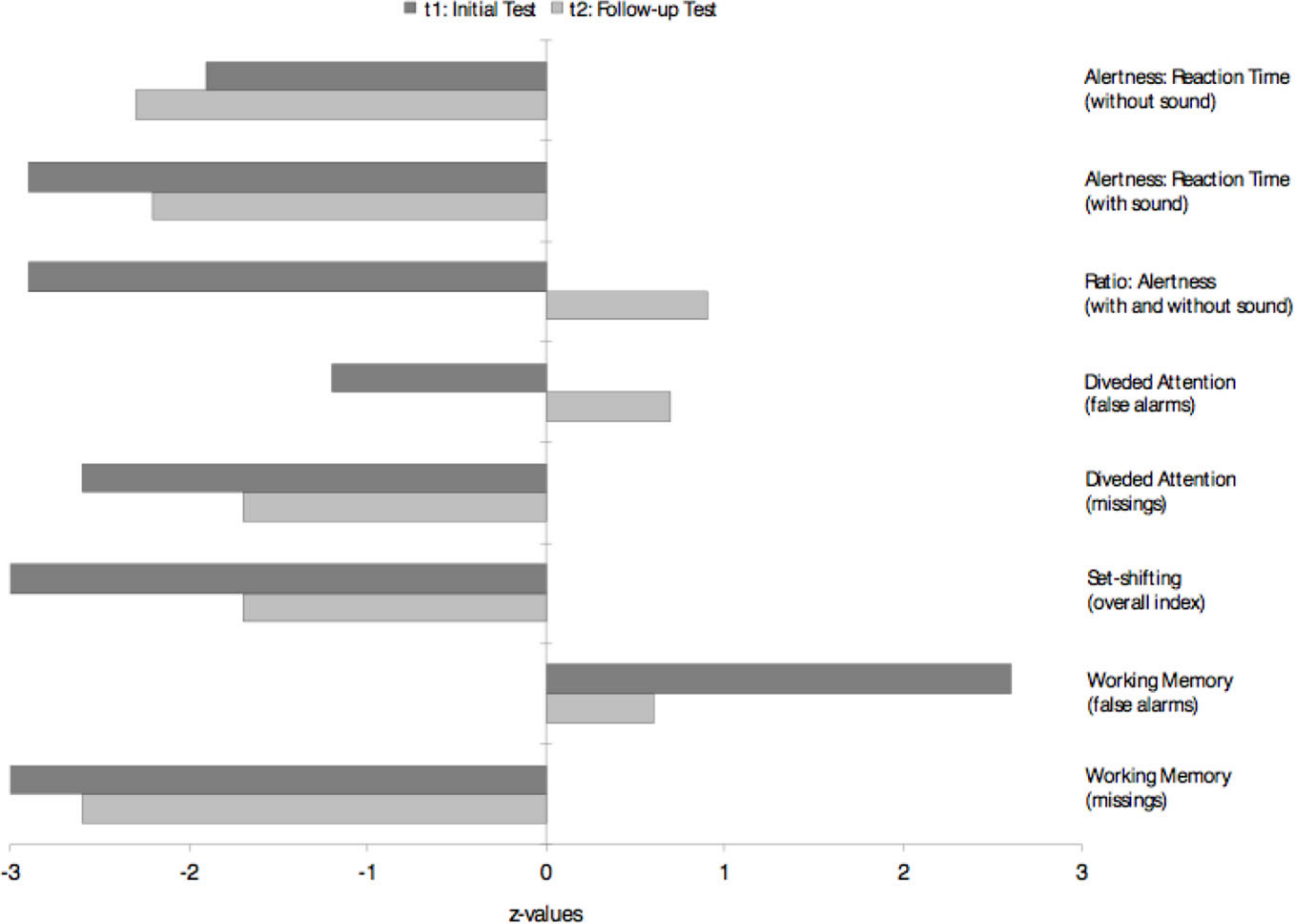
Comparison between initial (2010) and follow-up (2019) neuropsychological findings. The findings predominantly improved, but remain mainly still below average.

The first anti-NMDA-R antibody follow-up analysis in the reference laboratory in Oxford using a live cell-based assay (CBA) was still slightly positive after initial anti-inflammatory treatment (27 months after symptom onset). Several serological follow-up screenings (38, 48, 82, and 94 months after symptom onset) for IgG antibodies against neuronal cell surface antigens (AMPA-R, DPPX, GABA-B-R, LGI1, Caspr2, NMDA-R) using fixed CBAs showed normal anti-NMDA-R and other antineuronal antibody findings. Tests for anti-aquaporin 4 (AQP4) IgG and anti-myelin oligodendrocyte glycoprotein (MOG) IgG antibodies, possibly occurring when a demyelinating disorder (e.g., neuromyelitis optica spectrum disorder) develops after anti-NMDA-R encephalitis (2), were negative. Antibodies against intracellular antigens including SOX1 (initially slightly positive) were negative. The automated analysis of the structural MRI revealed an enlargement of the lateral ventricles with emphasis on the posterior horns and slight striatal and insular atrophy (**Figure 2**). There was no relevant change in findings between 2010 and 2019. A few new, but non-specific right-frontal white matter (WM) lesions were detected in the follow-up MRI. Visual EEG analyses depicted normalized findings; however, ICA detected left-side spike-wave activity and intermittent rhythmic delta activity (IRDA) as a correlate of remaining network instability. Overall, there was an increase in IRDA rates (**Figure 3** and **Table 1**). Until today, the gynecological screening for teratoma remains negative; initially, there was no evidence for an infection such as herpes encephalitis, and therefore, the trigger for anti-NMDAR encephalitis remains unclear. Compared with the initial neuropsychological testing, the authors detected improved attentional performance at follow-up testing using the Test for Attentional Performance (TAP) battery (Version 2.3.1; **Figure 4**).

**Table 1:** Diagnostic findings, initially (2009/10) and at follow-up (2019).

Investigation	Initial findings (2009/2010; [9])	Follow-up findings (2019)
Basic blood analyses	Glutamate pyruvate transaminase (GPT) was elevated (70 U/l; reference 10–35 U/l), γ-glutamyl transferase (γ-GT) was elevated (72 U/l; reference 0–40 U/l), other liver values were normal. Thyroid-stimulating hormone was elevated (4.77 µU/ml; reference 0.27–4.20 µU/ml), triiodothyronine, and thyroxine levels were in normal range.	Normal liver values. Thyroid-stimulating hormone, triiodothyronine, and thyroxine levels were in normal ranges. Vitamin B_12_/D, folic acid and selenium were normal.
Antibody findings	Screening for antibodies against neuronal cell surface antigens showed IgG antibodies against the NMDA-R (NR1-subunit; in the reference laboratory in Oxford using a live cell-based assay). Antibody against SOX1 were non-specifically slightly positive. Autoantibodies against TSH-receptor (TRAK) were elevated (4.77 µU/ml; reference 0.27–4.20 µU/ml), autoantibodies against thyroglobulin and thyroid peroxidase were normal. No screening for other immunological/rheumatological alterations was conducted.	Antibodies against different neuronal cell surface antigens (*AMPA-R, DPPX, GABA-B-R, LGI1, Caspr2*, and *NMDA-R*) were negative in serum (using biochip-assays from Euroimmun^®^). No antibodies against the intracellular onconeural antigens Yo, Hu, CV2/CRMP5, Ri, Ma1/2, SOX1, Tr(DNER), Zic4, or the intracellular synaptic antigens GAD65/amphiphysin were found (using Ravo line assay^®^). Autoantibodies against thyroglobulin, TSH receptor and thyroid peroxidase were not increased. Screening for antinuclear antibodies (ANA) showed a slightly positive homogenously result against nucleus and chromosomes (HEp-2), AMA/LKM, and anti-DFS70 were borderline positive (+). Anti-neutrophil cytoplasmic antibodies, antiphospholipid antibodies, rheumatoid factor, and anti-mitochondrial antibodies were negative. CH50 was slightly increased (131, reference: 65-115%), no other changes in the complement system (C3, C4, CH50, C3d) were observed. Normal serum IgA, IgM und IgG immunoglobulin concentrations; immunofixation showed no monoclonal antibody production. Anti AQP4-IgG and MOG-IgG antibodies were negative.
Cerebrospinal fluid analyses	Initially slight pleocytosis (23 µl; reference <5/µl). In the course normal white blood cell count (1/µl; reference <5/µl). Slightly elevated protein concentration (561 mg/L; reference <450 mg/L), and elevated age-corrected albumin quotient: 8.7; age-dependent reference <6.5 × 10^−3^) No CSF-specific oligoclonal bands; IgG index not increased (0.53; reference ≤0.7).	No lumbar puncture was conducted.
Cerebral magnetic resonance imaging with combined volume- and region-based analysis method (CVR) analysis	Inconspicuous findings, especially for the hippocampal regions and in the structures of the limbic system. Enlargement of the lateral ventricles with emphasis on the posterior horns and slight striatal and insular atrophy.	Except for a few non-specific right-frontal white matter lesions, the findings were essentially unchanged.
Electroencephalography – visual assessment	Intermittent delta focus over the right central areas.	Occipital α-activity (11 Hz).
Independent component analyses	1) Right and left frontotemporal delta waves; 2) a deep right temporal generator; and 3) a central component with theta frequencies.	Left-side spike-wave activity and intermittent rhythmic delta activity.
[^18^F]fluorodeoxyglucose positron emission tomography	Global cortical hypometabolism of the left hemisphere and right-temporal accentuation was detected. Cerebellar hypometabolism predominantly on the right side (most likely indicating crossed cerebellar diaschisis).	Not performed.
Cardiovascular examinations	Inconspicuous resting electrocardiography. Inconspicuous transthoracic echocardiography.	Inconspicuous resting electrocardiography. Raised long-term blood pressure.
Neuropsychological testing	Slower reaction times with evidence for heightened irritability and severely impaired ability to increase attention. Considerable amount of missings and errors in divided attention task. Severe deficits in cognitive flexibility. Considerable amount of missings and errors in working memory task	Slower reaction times with retained ability to increase attention. Considerable amount of missings and mean level of errors in divided attention task. Moderate deficits in cognitive flexibility. Considerable amount of missings and mean level of errors in working memory task

## Discussion

The authors report the case of a female patient with a protracted course of anti-NMDA-R encephalitis with, remarkably, one of the longest follow-up studies (approximately 10 years) to date focusing on long-term psychiatric symptoms.

### Long-Term Course—Process of Recovery

Based on present pathophysiological understanding, in our patient the anti-NMDA-R antibodies should have led to a receptor internalization in the forebrain and hippocampus (8) and thus to impairment of long-term synaptic plasticity (6, 8). The process of recovery can be described as a reversal of the period of illness and requires normally a longer hospitalization period (5, 10). During this process, inflammatory changes in diagnostic examinations mostly normalize after anti-inflammatory treatment (2). In the presented patient, the FDG-PET results were normalized five months later (9). In general, detected antibodies in CSF show a progressive decrease with symptoms resolving, which can take more than 18 months (4); low titers can persist for many months after recovery (11). In the present patient, the serum antibody findings were first reexamined seven months after first positive antibody detection and after completed anti-inflammatory acute therapy; at that time, they were still slightly positive, and since then have always been negative (but using a different methodology of antibody testing). Follow-up monitoring of antibody titers revealed an imperfect correlation with clinical course (2, 3). Therefore, clinical assessment of the patient is still the primary source of evidence when considering maintenance versus tapering of therapy (2, 3). At 24-month follow-up, the largest cohort study by Titulaer et al. identified 45 of 577 clinical relapses mostly presenting as milder and predominantly mono-symptomatic syndromes compared to the initial presentation (4). Following return to baseline functioning, the majority of patients (85%) still experience significant cognitive and behavioral abnormalities requiring supervision and rehabilitation (5). Neuropsychological residua, particularly impaired processing speed or episodic memory, persist with a significantly higher prevalence when the initiation of immunotherapy was delayed (12). The late onset of treatment in the present patient may have contributed to the persistence of neurocognitive symptoms. Another long-term observation of 24 months in a pediatric patient discerned sustained symptom relief (13). Moreover, sufficient symptom relief had been achieved, for example, in a case of a 24-year-old male patient under anti-inflammatory treatment (intravenous immunoglobulins, steroids) after a protracted course of 4 years that included episodes of catatonia, diffuse theta slowing in EEGs, and CSF pleocytosis (14). Another case report of a 22-year-old female patient described, for example, a more severe relapse of anti-NMDAR encephalitis five years after the initial episode responding well to second-line immunotherapy (cyclophosphamide) (15). Symptoms in the presented patient were dominated by a persistent dysexecutive syndrome with 1) intense sensory overload, 2) cognitive deficits, and 3) fatigue and reduced energy level, as well as sleep disturbances, as already described in other cases with shorter courses (2, 5, 7, 16). A characteristic persistent amnesia of the entire acute phase of illness (6) also existed in the present case.

### Clinical Implications

The current patient showed a remarkable response to low-dose atypical neuroleptics (initially risperidone 2 mg/day, later amisulpride 200 mg/day). Both substances strongly reduced the level of sensory overload and normalized the reduced energy levels. This positive effect of low-level neuroleptics is comparable to the symptom relief of patients with autism spectrum disorders treated with low-dose antidopaminergic medication (17). In this particular constellation, it is important to keep in mind that patients with acute anti-NMDA-R encephalitis more frequently develop side effects of neuroleptics up to the occurrence of a neuroleptic malignant syndrome (10); therefore, doses should be started low and increased slowly. In fact, the present patient developed a severe psychomotor restlessness under just 2.5 mg/day aripiprazole. From a psychotherapeutic perspective, states of sensory overload are often associated with experiences of stress and anxiety. Thus, identifying and reducing stressful stimuli as well as applying adaptive coping strategies are central aims of the treatment. The applicability of coping strategies should be evaluated and trained in various situations to regain control over daily life. In addition, to gain cognitive improvement by cognitive training, the acceptance of cognitive limitations is an important treatment focus in case of enduring cognitive impairments. Enrolling patients with ongoing psychiatric deficits after acute anti-NMDA-R encephalitis in a multidisciplinary setting over the long-term, including psychopharmacotherapy, CBT with psycho-education, and close neuropsychological monitoring, could probable achieve positive outcomes (16).

The EEG findings about 10 years later showed clear alterations (spike-wave complexes and a higher rate of IRDAs, both on the left side) and can be interpreted as a neuroinflammatory scar in the absence of current signs of active neuroinflammation. The left hemispherical localization of the spike-wave complexes in the current EEG is congruent with the localization of hypometabolism on the initial FDG-PET (9). According to the concept of the local area network inhibition (LANI) hypothesis (18), the dysexecutive symptoms could be caused by neuronal network hyperinhibition; therefore, the neuroleptic treatment could have led to the breakthrough of hyperinhibition. Alternatively, a reduction of the underlying excitation by e.g., anticonvulsants might be also helpful in similar cases (18), and was also initially tried with levetiracetam in the patient.

### Limitations

It is important to mention that the authors have presented the course of just a single patient suffering from anti-NMDA-R encephalitis. The initial diagnostic clarification after 21 months led to a delayed start of treatment. The long untreated course may have enhanced the ongoing dysexecutive syndrome, as it is well known that late treatment is associated with poorer prognosis (4, 6). A full remission can also be achieved in patients with a short course of disease (19). In the present patient, the symptomatic psychiatric treatment with low-dose, atypical neuroleptics and psychotherapy was also started late. The authors believe that, in future, patients with persistent dysexecutive symptoms should be treated continuously in a multidisciplinary manner enabling the start of early symptomatic treatment.

## Conclusions

In summary, the case reported highlights the importance of long-term observation and a multidisciplinary approach in treating patients with anti-NMDA-R encephalitis. Follow-up descriptions of larger cohorts discussing psychiatric residual symptoms and their treatment over the long term are necessary. Treatment with neuroleptics and CBT could play an important role in similar cases.

## Author’s Note

This is the follow-up to an earlier case report (9).

## Data Availability Statement

All necessary information is included in the article.

## Ethics Statement

The described patient gave her signed written informed consent for this case report to be published, including the publication of any potentially identifiable images and all data included in this article.

## Author Contributions

DE, LT, DD, TS, and EP treated the patient. SM performed the data research. SM and DE wrote the paper. BF performed the EEG analyses and interpreted the EEGs. TS performed the psychotherapy and did the neuropsychological testing. HU interpreted the MRIs. NV performed the immunological measurements and interpreted the results. HP supported the neurological interpretation. LT, KR, KN, SJM, MM, and KD supported clinical interpretation. All authors were involved in the theoretical discussion and composition of the manuscript. All authors read and approved the final version of the manuscript.

## Funding

The article processing charge was funded by the German Research Foundation (DFG) and the University of Freiburg in the funding program Open Access Publishing.

## Conflict of Interest

LT: Advisory boards, lectures, or travel grants within the last 3 years: Roche, Eli Lilly, Janssen-Cilag, Novartis, Shire, UCB, GSK, Servier, Janssen and Cyberonics. KD: Steering Committee Neurosciences, Janssen. HU: Shareholder of the Veobrain GmbH.

The remaining authors declare that the research was conducted in the absence of any commercial or financial relationships that could be construed as a potential conflict of interest.
